# Design and Control Method of Passive Energy Harvesting for Hydropower Unit Sensors in Complex Electromagnetic Environments

**DOI:** 10.3390/s26092628

**Published:** 2026-04-24

**Authors:** Xiaobo Long, Zhijun Zhou, Zhidi Chen, Peng Chen

**Affiliations:** 1 China Yangtze Power Co., Ltd., Yichang 430014, China; 2 Hubei Technology Innovation Center for Smart Hydropower, Wuhan 430014, China; 3 CTG Wuhan Science and Technology Innovation Park, Wuhan 430014, China

**Keywords:** magnetic energy harvesting, non-intrusive micro-energy harvesting, maximum power point tracking, genetic algorithm

## Abstract

With the advancement of digital hydropower stations, the requirements of real-time, high-precision industrial soft measurement of key power equipment operating status are attracting more and more attention. However, it is difficult to transfer energy to the monitoring sensor in strong electromagnetic environments. In this paper, a high-efficiency, high-power-density magnetic field energy harvester is proposed for monitoring sensors in hydropower stations, which captures the energy from the magnetic flux leakage of a hydroelectric generating set. Efficient magnetic energy capture is achieved by modeling material properties and optimizing the receiver’s magnetic core parameters via a Genetic Algorithm. The theoretical analysis of charging characteristics is given, and a Maximum Power Point Tracking (MPPT) control circuit is proposed, realizing high-efficiency energy conversion. Finally, an experimental planet is built. Under 70–130 Gs power-frequency magnetic fields, the system delivers 2.8–5.1 V open-circuit voltage, 66 mW maximum load power, and 6.5 mW/cm^3^ power density.

## 1. Introduction

The intelligent development of hydropower units is driven by smart power stations and uncrewed monitoring technologies. Real-time, high-precision industrial soft sensing is required for the stator core. The stator core is defined as the core magnetic circuit of the unit. the safe operation of hydropower plants is guaranteed by this measurement technology. Early fault detection, cost reduction, and efficiency improvement are realized by data-driven condition awareness [1,2,3,4,5,6]. However, conventional installation and maintenance processes are challenged by the intense electromagnetic environment of hydropower units. Significant power supply challenges are posed for the matched monitoring sensor systems. Wiring difficulties, high costs, and potential safety risks are presented by traditional power supply methods (e.g., wired power supply). Similarly, inconvenient maintenance and short service life are exhibited by battery-powered systems. Requirements for long-term stable monitoring are not satisfied by these schemes [7,8,9,10,11,12].

These power supply limitations are effectively addressed via energy harvesting from ambient sources (vibration, heat, and electromagnetic fields). This approach is recognized as a promising solution for wireless sensor networks (WSNs). The critical role of self-powered technologies in the Industrial Internet of Things (IIoT) has been widely highlighted by recent studies. This technology is deemed particularly valuable for the monitoring of remote or hazardous assets [13]. Among these available sources, the electromagnetic environment in hydropower plants is widely regarded as an ideal energy reservoir. Higher stability and energy density are provided by this environment, compared with vibration or thermal energy sources [14].

Currently, magnetic field energy harvesting (MFEH) technologies for power systems are broadly classified into two types: current transformer (CT)-based and free-standing structures. CT-based harvesters are clamped around transmission cables. These devices are extensively investigated for power line monitoring applications [15,16,17,18]. A closed magnetic circuit is adopted to achieve high energy transfer efficiency. However, this “clamp-on” structure is deemed fundamentally unsuitable for hydropower unit stator cores. Large, flat ferromagnetic surfaces are presented by these stator cores, and no accessible cables for encirclement are provided by these core structures.

Consequently, free-standing or non-intrusive harvesters utilizing leakage flux are given increasing research attention. Various core geometries are investigated to improve the energy capture density of these open-magnetic-circuit devices. For instance, a coreless structure is presented in Reference [19] to reduce device weight, and a compact rectangular core is developed in Reference [20]. More complex structures (e.g., “X” shape [21], “H” shape [22,23]) are proposed to optimize magnetic flux concentration.

Furthermore, a cascaded magnetic circuit structure is innovatively proposed in References [24,25]. Energy density is significantly enhanced by this structure. Meanwhile, the influence of core parameters has been systematically analyzed in related studies [26,27]. Corresponding optimal design criteria are established based on the above analyses.

Despite these advancements, a critical research gap is still identified. Most existing designs are focused on high-frequency environments or general transmission lines. These designs are applied to hydropower units. The hydropower units are characterized by 50 Hz low-frequency and high-intensity leakage flux (70–130 Gs). Low effective permeability and insufficient power density are often exhibited by these designs under this operating condition. The root cause of this performance issue is clarified by Faraday’s law of electromagnetic induction. The induced voltage of the harvester is confirmed to be proportional to the excitation frequency. A sharp drop in energy capture capability at 50 Hz power frequency is caused by this proportional relationship. Meanwhile, conventional designs are mostly developed based on empirical single-parameter tuning. The demagnetization coefficient and flux concentration capability of the core are not effectively optimized by these designs for low-frequency application scenarios. Further exacerbation of power density degradation is induced by this design limitation.

Additionally, efficient power management is critical for practical application. Inefficiency is induced in direct coupling by impedance mismatch. This feature is emphasized by recent works [28]. Meanwhile, the critical application value of energy harvesting technology in power equipment defect sensing and fault monitoring is further validated by recent studies [29,30]. The feasibility of energy harvesting technology to realize self-powered, long-term stable condition monitoring for industrial equipment is verified by these research works. The development prospects of the self-powered sensing technology for hydropower unit condition monitoring are fully confirmed by these research findings. This sensing technology is proposed in this work. Advanced Maximum Power Point Tracking (MPPT) circuits are necessitated to handle fluctuating magnetic field energy.

To address these issues, a high-efficiency magnetic field energy harvesting system design and corresponding control method are proposed in this paper. Prior free-standing H-shaped core harvesters are mostly developed for transmission line or high-frequency application scenarios. Local optimal parameter tuning is realized by these harvesters through univariate scanning. Different from these prior designs, material parameter modeling is integrated with a Genetic Algorithm (GA) in this work. The H-shaped magnetic core structure is globally optimized by this combined method. Maximum magnetic flux capture is guaranteed under the specific constraints of hydro unit stator application scenarios.

Compared with similar schemes, the installation limitation of CT-type harvesters is effectively avoided by the proposed design. Encircling cables are required by traditional CT-type harvesters. Higher low-frequency power density is achieved by this design, compared with coreless, rectangular, and X-shaped free-standing harvesters. The impedance mismatch problem is solved by the customized MPPT circuit. Energy conversion efficiency is effectively improved by this circuit design. This design is specially adapted to the 50 Hz, 70–130 Gs power-frequency leakage magnetic field of hydropower environments. Non-intrusive surface mounting is supported by this design. The modification of the original hydro unit is not required by this mounting scheme. Excellent mechanical stability is exhibited by the proposed design. The long-term vibration of hydro units is well adapted by this stable structure.

Furthermore, a customized MPPT control circuit is developed to regulate weak and fluctuating energy output. Key parameters of the magnetic core and coils are systematically optimized. Efficient magnetic field capture, coordinated energy regulation, and dynamic scheduling are realized by the proposed system. Stable power support for generator condition monitoring is provided by this integrated system. The engineering implementation of industrial soft measurement technology in smart power plants is effectively promoted by this technical scheme.

## 2. Modeling and Analysis of Non-Invasive Magnetic Field Energy Harvesters

### 2.1. Energy Extractor Structural Design

A non-closed structure is adopted for the magnetic core of non-invasive magnetic field energy harvesting. The core is subjected to an external magnetic field, and magnetization is induced. Magnetic poles are formed at its two ends. These poles generate a magnetic field within the core—this field opposes the external field *H_ex_* [31,32,33]. It is referred to as the demagnetizing field, with an intensity of *H_d_*. The magnetic field strength *H_in_* within the core is weakened by this demagnetizing field. This relationship is expressed as:

(1) $$H_{\mathrm{in}}\;=\;H_{\mathrm{ex}}-H_{d}.$$

The demagnetizing field strength *H_d_* is correlated with the magnetization intensity *M* as follows:

(2) $$H_{d}\;=\;M\times D_{m}.$$

In the formula, *D_m_* is denoted as the demagnetization coefficient.

A weaker demagnetizing field is induced by a smaller demagnetization coefficient, and a stronger internal magnetic field is generated within the core by this coefficient. The demagnetization coefficient is determined by the structure and dimensions of the magnetic core, and the coefficient is effectively reduced by a slender core structure. A symmetrical structure is adopted for the H-shaped core, the magnetic circuit is constrained by laminations at both ends of the core, magnetic leakage is effectively reduced by this design, and the magnetic concentration capability of the core is significantly enhanced. High winding space utilization is provided by the core’s central column, a low demagnetization coefficient is achieved by the slender structure of the central column, and convenient installation is realized by this overall configuration. Compared with other common core geometries, higher magnetic flux concentration is achieved by the proposed core. Superior flux concentration performance is obtained versus coreless and rectangular cores. Better winding adaptability and mechanical stability are exhibited under hydropower unit vibration environments compared with complex X-shaped cores. Thus, the H-shaped core structure is selected for further analysis and research.

**Figure 1:**
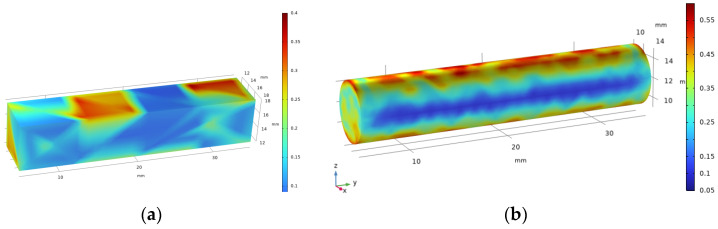
Different-shaped magnetic flux density distribution plots: (**a**) square; (**b**) round.

Magnetic flux concentration capabilities are affected by different shapes of the central column. Simulation comparisons are conducted under a 50 Gs power-frequency magnetic field. In engineering applications, typical central column shapes are defined as square and circular structures. Convenient processing and winding are realized by the square structure. Superior magnetic flux concentration performance and assemblability are provided by the circular structure. These two structures are selected for comparative analysis. The corresponding simulation results are presented in Figure 1.

As shown in Figure 1, compared with square core structures, cylindrical core structures have higher magnetic flux density at the central column. This enhances the magnetic concentration capability. Thus, a magnetic field energy harvesting device with a circular central column is designed.

### 2.2. Energy Harvester System Modeling and Power Density Analysis

**Figure 2:**
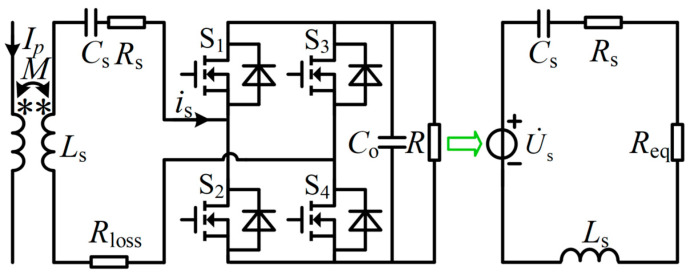
Non-intrusive magnetic field energy harvester equivalent circuit model.

The equivalent circuit model of a non-intrusive magnetic field energy harvester is shown in Figure 2. In the figure, the core current of the hydro turbine unit is represented by *I_p_*; the magnetic coupling mutual inductance of the energy harvesting device is denoted by *M*; the self-inductance of the coil is represented by *L_s_*; the compensation capacitance is denoted by C_s_; the internal resistance of the coil is represented by *R_s_*; the core loss is denoted by *R_loss_*; the rectifier bridges are labeled *S*_1_ to *S*_4_; the output filter capacitance is represented by *C_o_*; the load is denoted by *R*; the equivalent DC load is represented by *R_eq_*.

The law of electromagnetic induction is applied to determine the magnitude of the induced voltage *U_s_* in the coil. It is expressed as:

(3) $$U_{s}\;=\;\omega NBA\mu_{\mathrm{eff}}.$$

In the equation, the magnetic flux density in the air domain of the current-carrying conductor is represented by *B*; the magnetic core area enclosed by the coil is denoted by *A*; the effective magnetic permeability of the core is represented by *μ_eff_*.

System transmission characteristics are improved, and reactive power losses are reduced. For this purpose, a resonant state should always be maintained between the compensation capacitance and the coil’s self-inductance. This state is expressed as:

(4) $$C_{s}\;=\;\frac{1}{\omega^{2}L_{s}}.$$

In the equation, the resonant frequency is represented by *ω*.

The system compensation element is made to satisfy Equation (4). Under this condition, the system output voltage is determined as:

(5) $$U_{o}\;=\;\frac{8RU_{s}}{\pi^{2}\left(R_{s}+\frac{8}{\pi^{2}}\right)}.$$

The output power of the system’s equivalent load requirement is derived from the above equation. It is expressed as:

(6) $$P\;=\;\frac{8RU_{s}^{2}}{\pi^{2}{\left(R_{s}+\frac{8}{\pi^{2}}\right)}^{2}}.$$

The power density of the system is measured using a specific definition. The ratio of output power *P* to core volume *V_EH_* is defined as power density *ρ*. This definition is:

(7) $$\rho \;=\;\frac{8RU_{s}^{2}}{\pi^{2}{\left(R_{s}+\frac{8}{\pi^{2}}R\right)}^{2}V_{EH}}.$$

In the equation, the core volume is represented by *V_EH_*.

Equation (7) shows key factors affecting the system’s power density. The system’s power density can be effectively enhanced by increasing the coil’s induced voltage, reducing the coil’s parasitic resistance, and decreasing the core volume.

**Figure 3:**
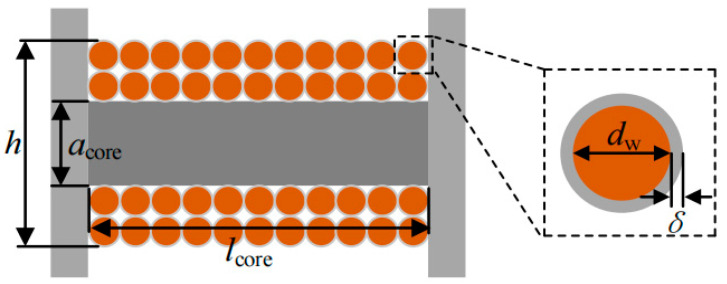
Winding cross-section schematic.

A schematic cross-section of the core coil is shown in Figure 3. Its winding length *l_core_* is determined by the magnetic column side diameter *a_core_* and the coil domain diameter *h*. The number of coil turns *N* is expressed as follows:

(8) $$N\;=\;\frac{\left(h-a_{\mathrm{core}}\right)l_{\mathrm{core}}}{2{\left(d_{w}+2\delta \right)}^{2}}.$$

In the formula, the wire diameter of the coil is denoted by *d_w_*; the thickness of the insulation layer is denoted by *δ*.

The magnetic flux density *B* generated by a current-carrying conductor in an air domain is approximately calculated as:

(9) $$B\;=\;\frac{\mu_{0}I_{p}}{2\pi \lambda }.$$

In the equation, the permeability of free space is represented by *μ*_0_; the perpendicular distance from the energy harvester to the current-carrying conductor is denoted by *λ*.

The cross-sectional area *A* of the magnetic core enclosed by the coil is determined as:

(10) $$A\;=\;\pi a_{\mathrm{core}}^{2}.$$

Thus, the induced voltage can be derived and determined as:

(11) $$U_{s}\;=\;\frac{\omega \mu_{0}\mu_{r}Il_{\mathrm{core}}a_{\mathrm{core}}^{2}\left(h-a_{\mathrm{core}}\right)}{4\lambda {\left(d_{w}+2\delta \right)}^{2}\left[1+D_{m}\left(\mu_{r}-1\right)\right]}.$$

In the equation, the relative magnetic permeability of the core is denoted by *μ_r_*.

Meanwhile, the coil resistance *R_s_* is given by the following equation:

(12) $$R_{s}\;=\;\frac{\rho_{0}}{\pi {\left(\frac{d_{w}}{2}\right)}^{2}}\frac{4\rho_{0}\left(h^{2}-a_{\mathrm{core}}^{2}\right)l_{\mathrm{core}}}{\pi d_{w}^{2}{\left(d_{w}+2\delta \right)}^{2}}.$$

In the formula, the coil resistivity is represented by *ρ*_0_.

For an energy harvester based on an H-shaped structure, the coil is wound around the central magnetic column. Therefore, the volume *V_EH_* of the energy harvester is calculated as:

(13) $$V_{\mathrm{EH}}\;=\;\pi \left(r^{2}-a_{\mathrm{core}}^{2}\right)l_{\mathrm{core}}+bD^{2}+\pi a_{\mathrm{core}}^{2}l.$$

Thus, the power density of the energy harvester is given by the following equation:

(14) $$\rho \;=\;\frac{R\omega^{2}\mu_{0}^{2}\mu_{r}^{2}I_{p}^{2}l_{\mathrm{core}}^{2}\left(h-a_{\mathrm{core}}\right)a_{\mathrm{core}}^{4}d_{w}^{4}}{A*B}.$$

In the equation, A = $32\lambda^{2}V_{\mathrm{EH}}^{2}{\left[1+D_{m}\left(\mu_{r}-1\right)\right]}^{2}$,

*B* = ${\left[\pi \rho_{0}l_{\mathrm{core}}\left(h^{2}-a_{\mathrm{core}}^{2}\right)+2Rd_{w}^{2}{\left(d_{w}+2\delta \right)}^{2}\right]}^{2}\;$.

A close relationship between the device’s power density and multiple key parameters is revealed by the above equation. These parameters are specified as effective magnetic permeability, coil resistivity, core dimensions, and coil turns. Therefore, the power density of the device is effectively enhanced by adjusting the above device parameters.

## 3. Optimization Design and Research of Energy Harvester Magnetic Core and Coil Parameters

### 3.1. Magnetic Core Material Design

The effective permeability of the core is decisively influenced by the relative permeability of core materials. The relative permeabilities are defined as *μ_r_*_1_ = 1 and *μ_r_*_2_. The effective magnetic fluxes are defined as *φ*_1_ and *φ*_2_, respectively. The magnetic flux in the core is given by:

(15) $$\left\{\begin{array}{c} \varphi_{1}\;=\;\mu_{0}HS\\ \varphi_{2}\;=\;\mu_{eq}\mu_{0}HS \end{array}\right..$$

Therefore, the effective magnetic permeability is:

(16) $$\mu_{eq}\;=\;\varphi_{1}/\varphi_{2}.$$

**Figure 4:**
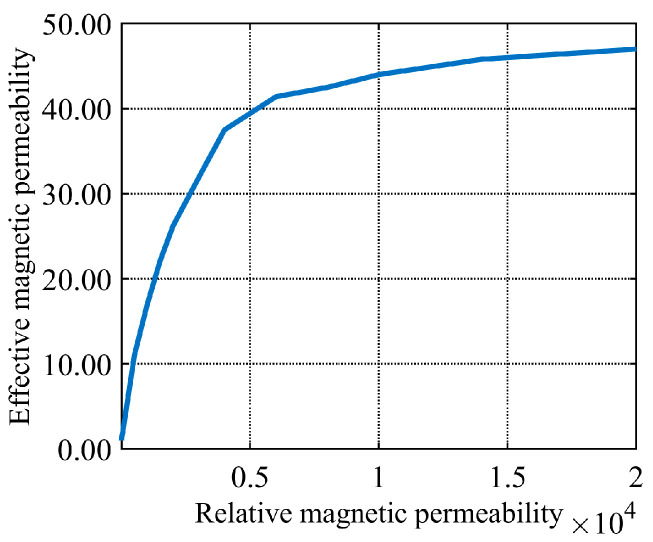
Effective and relative permeability curves.

The variation of relative permeability versus effective permeability for different core materials is presented in Figure 4. A relative permeability higher than 4000 is observed for the core material from this figure. Under this condition, the effective permeability of the H-shaped core is gradually rendered less sensitive to changes in relative permeability. Therefore, a core material with relative permeability greater than 4000 is required for the core design. This relative permeability requirement is satisfied by silicon steel. Advantages of high magnetic saturation, high resistivity, and low cost are also provided by this material. Consequently, silicon steel is selected as the core material in this study.

### 3.2. MPPT Magnetic Core Parameter Design and Optimization

**Figure 5:**
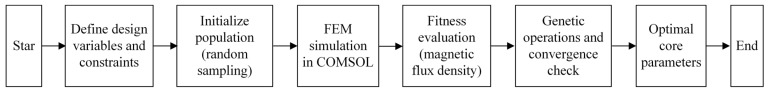
Flowchart of the magnetic core structure optimization based on magnetic flux density.

The magnetic flux concentration capability of an H-shaped magnetic core is significantly affected by its structural dimensions. From the perspective of the physical mechanism, the magnetic flux path is constrained by the side plates of the H-shaped core. External leakage flux is effectively reduced by this structural design. Ambient magnetic flux is guided to converge into the central column of the core. An effective magnetic path constraint is not formed by undersized side plates. Local magnetic saturation of the core is induced by oversized side plates. The effective permeability of the core is reduced by this saturation effect. The flux concentration capability is weakened accordingly. According to the magnetic circuit principle and the demagnetization model (Equations (1) and (2)) in this work, the flux path is regulated by side plate dimensions. This regulation is achieved by changing the magnetic boundary constraint and demagnetization coefficient *D_m_*. Local saturation at the junction of the side plate and central column is induced by oversized side plates. The effective permeability of the core is sharply reduced by this local saturation (Equations (15) and (16)). The magnetic reluctance of the magnetic circuit is increased simultaneously. The low-reluctance flux guidance path is blocked by this effect. To optimize the power density and output power of non-invasive energy harvesting devices, a COMSOL simulation model is established for an H-shaped magnetic core energy harvesting device. The effects of the central column length *l*, diameter *a*, central column panel thickness *b*, and side panel edge length *D* on the magnetic flux density within the central column are analyzed. The variation curves of the column magnetic flux density versus these parameters (central column length, central column diameter, side panel thickness, side panel edge length) are shown in Figure 5. These curves are obtained in a 50 Gs power-frequency magnetic field environment.

The magnetic flux concentration capability of an H-shaped magnetic core is significantly influenced by its dimensions. To optimize the power density and output power of non-invasive energy harvesting devices, the effects of the key parameters are analyzed. These parameters include the central column length *l*, diameter *a*, central column plate thickness *b*, side plate width *w*, and side plate height *h*. They affect the internal magnetic flux density of the central column. This analysis is based on a COMSOL simulation model. The model is built for an energy harvesting device utilizing an H-shaped magnetic core. Under external alternating magnetic field conditions, the average magnetic flux density in the core’s central column region is denoted as *Bc*. The central column’s magnetic flux density is selected as the objective function for core structure optimization. The optimization model is expressed as:

(17) $$\underset{x}{max}B_{c}\left(x\right).$$

Among these, the design variable vector for the magnetic core structure is defined as:

(18) $$x\left[l,a,b,w,h\right].$$

where *l* is the central column length, *a* is the central column diameter, *b* is the side plate thickness, *w* is the side plate width, and *h* is the side plate height.

The installation space, electromagnetic performance, and engineering manufacturability of the magnetic core are considered, and the following constraints must be satisfied during the optimization process:

(19) $$B_{c}\left(x\right)\;\leq \;B_{sat}\;{xmax}_{min}$$

Among these, *B_sat_* is denoted as the magnetic saturation flux density of the silicon steel core material. *x_min_* and *x_max_* are determined by the actual installation and processing conditions, respectively. Specifically, the upper bound of *B*_*c*_ is constrained by the saturation flux density of 27Q100 silicon steel. Performance degradation is effectively avoided by this constraint. The upper bounds of the dimensional variable xmax are strictly limited by the 30 mm × 30 mm × 30 mm non-intrusive installation space around the hydro unit stator. The lower bounds of xmin are defined by the industrial cutting accuracy of silicon steel laminations and the minimum winding space for the 1000-turn coil. Standard industrial manufacturability is guaranteed by this boundary setting.

A significant nonlinear coupling relationship between the core structure parameters and the magnetic flux density in the central column is confirmed. Additionally, high dimensionality is exhibited by these parameters. The global optimal solution is not readily acquired through sole reliance on empirical selection or univariate scanning. Therefore, a combined approach of “finite element simulation + intelligent optimization algorithms” is utilized for core structure optimization.

First, random sampling of the core structure parameters is implemented. This sampling is conducted within the allowable range of design variables. A magnetic field finite element model is established via COMSOL software. Magnetic flux density in the central column region under different parameter combinations is calculated by this model. The average magnetic flux density in the central column region is set as the performance evaluation metric. A mapping dataset between the core structure parameters and magnetic flux density is constructed accordingly.

On this basis, a Genetic Algorithm (GA) is introduced. It is applied for the global optimization of magnetic core structural parameters. Compared with gradient-based optimization methods and univariate scanning methods, inherent advantages in gradient-free global optimization are exhibited by the Genetic Algorithm. Local optima are easily trapped by gradient-based optimization methods for this multi-variable nonlinear coupling problem. Parameter interaction is not accounted for by the univariate scanning method. This inherent advantage is identified as the core reason for the selection of GA. The randomness of crossover and mutation operations, combined with the elite retention strategy, is adopted. Local optima during the iteration process are effectively avoided by this combined strategy. During the optimization process, the average magnetic flux density of the central column is defined as the fitness function. Parameter combinations are iteratively updated through population initialization, selection, crossover, and mutation operations. In each iteration, the fitness of the candidate solutions is calculated based on finite element simulation results. Once convergence criteria are satisfied, the optimal structural parameter combination is output by the optimization algorithm. The magnetic flux density of the central column is maximized under specified constraints by this scheme. The pitfalls of traditional manual parameter tuning and single-parameter scanning are effectively avoided by this optimization method. Local optima are frequently induced by these traditional tuning approaches. The systematic optimization design of magnetic core structural parameters is realized by this proposed method. The specific optimization flowchart is presented in Figure 5.

**Figure 6:**
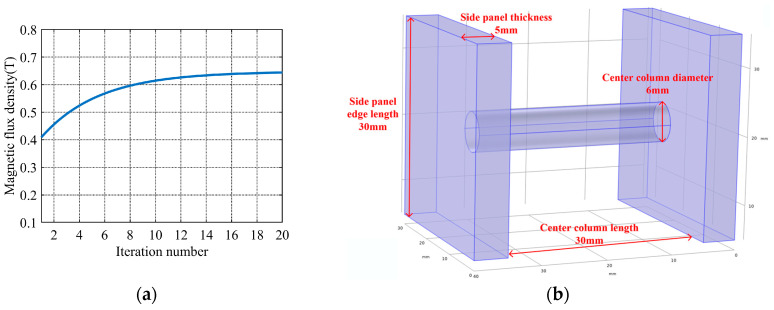
(**a**) Convergence curve of the magnetic flux density during the optimization process. (**b**) Optimal dimension parameters schematic.

The aforementioned method was adopted. After a finite number of iterations, the magnetic flux density gradually converged. This indicates that near-optimal structural parameters had been obtained, as shown in Figure 6a. The final optimal dimensional parameters of the magnetic core were determined: *l* = 30 mm, *a* = 6 mm, *b* = 5 mm, *t* = 30 mm, and *w* = 30 mm, as shown in Figure 6b. Simulation results are obtained by COMSOL simulation, and conventional H-shaped core parameters with the same overall size are set as the reference group. A 30–35% increase in the average magnetic flux density of the central column was achieved by GA-optimized parameters, and a corresponding 40–45% increase in the output power of the harvester was achieved by these optimized parameters. The performance gain brought by GA optimization was quantitatively verified by the above results.

### 3.3. Coil Parameter Optimization and Research

The core component for magnetic-to-electrical conversion in energy harvesting devices is the coil. Induced voltage and power density are enhanced by more coil turns. Excessive turns increase internal resistance, leading to higher system losses and reduced load capacity. Additionally, uneven magnetic field energy distribution affects output power from different coil installations. Thus, research on the influence of turns and position on coil output is needed. Coil displacement or loosening may be caused by unit vibration, so uniform geometric shape and winding count must be maintained.

**Figure 7:**
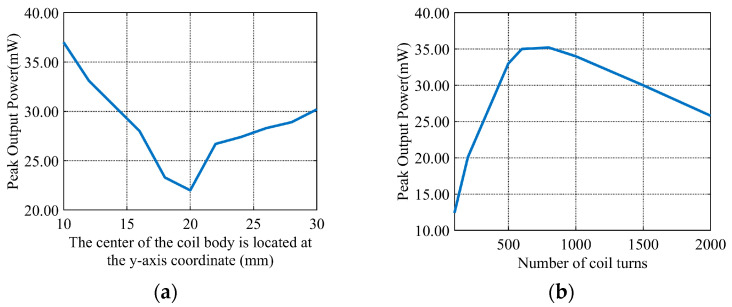
Output peak power vs. *y*-axis position and number of coil turn curves. (**a**) Position along the *y*-axis. (**b**) Number of coil turns.

The effect of the coil’s spatial position on peak output power is analyzed via simulations. Coil turns are kept constant, while the coil center’s position is varied along the *y*-axis. The results in Figure 7a show that peak output power is maximized when the coil is at either end of the side panel. With the coil position fixed, the transmission characteristics of different turn counts are analyzed. Simulations are conducted for 0–2000 turns, with the results shown in Figure 7b. Peak output power first increases then decreases with turns, with an optimal performance between 500 and 1000 turns. Induced voltage and output power are enhanced by increased coil turns. A significant rise in the coil’s parasitic resistance is caused by excessive turns. Higher ohmic loss and decreased power density are induced by the raised parasitic resistance. The peak output power of 66 mW is achieved at 1000 coil turns. The peak output power of 42 mW is obtained at 1500 coil turns. A 120% higher parasitic resistance and 28.3% lower power density are observed below the 1500-turn condition. Considering parasitic resistance and system losses, a 1000-turn coil is selected to balance output performance, power density, and losses.

## 4. Energy Management Circuit Design and Research

**Figure 8:**
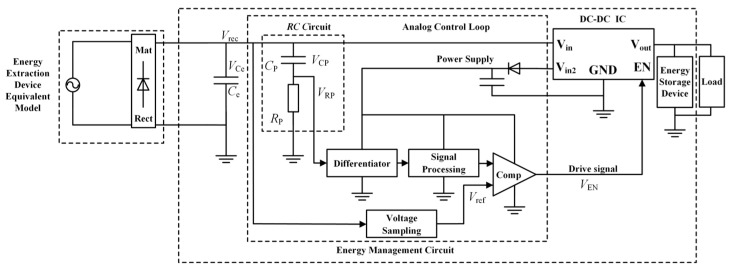
Energy harvesting system structure schematic.

The structure of the energy management circuit is shown in Figure 8. A series-resonant matching circuit and a quadruple voltage rectifier circuit are included in the front-end. A Maximum Power Point Tracking (MPPT) circuit is contained in the back end. This MPPT circuit incorporates an analog control loop and a DC–DC power supply chip.

### 4.1. Matching and Rectifier Circuits

The energy harvesting coil has an inductive nature. A portion of the harvested energy is consumed as reactive power. The output power of the harvesting device is reduced by this consumption. Therefore, a matching network is incorporated after the harvesting device to enhance output power. Resonance principles are utilized by this network. The inductive component in the coil’s output impedance is canceled through these principles. Consequently, a series resonant matching circuit is employed in this paper. A series capacitor *C* and coil inductance *L* are selected to achieve resonance. The output power is thereby increased.

Simultaneously, the alternating current (AC) voltage is output by the energy harvesting device. This AC voltage cannot directly power the sensing system. Consequently, an AC–DC conversion of the harvested output is required. A quadruple voltage-doubling rectifier circuit is employed in this paper. The voltage is rectified, and its level is elevated simultaneously by this circuit.

**Figure 9:**
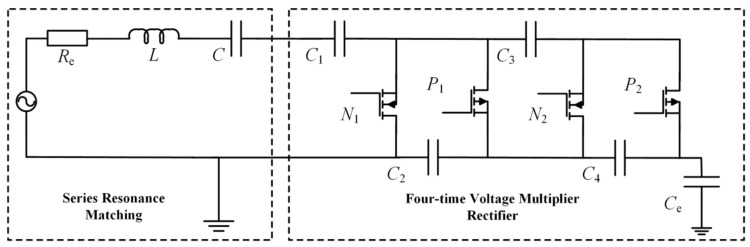
Matching and rectifier circuit structure schematic.

Furthermore, rectifier diodes are replaced by power MOSFETs. These MOSFETs have extremely low on-state resistance. The conduction voltage drop is significantly reduced. Rectification losses are minimized accordingly. The structure of the resonant matching and voltage-doubling rectifier circuit is shown in Figure 9.

### 4.2. MPPT Control Circuit

**Figure 10:**
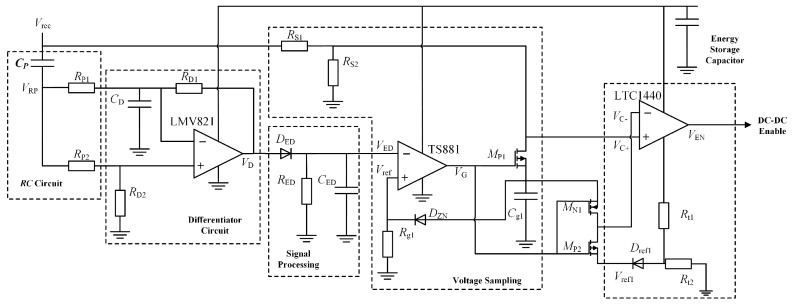
Control loop structure schematic.

An RC circuit, a differential circuit, a signal processing circuit, a voltage sampling circuit, and a comparator are included in the MPPT control circuit. The energy management circuit is enabled to track the maximum power output from the collector by this control circuit. The loop structure is shown in Figure 10. The peak voltage V_RP_ of the RC circuit’s output is detected by the differential circuit. A sharp pulse is generated by the differential circuit simultaneously. This sharp pulse is smoothed into a continuous signal by the signal-processing circuit (envelope detection). The rectified output *V_rec_* is stepped down to the reference voltage *V_ref_* by the voltage sampling circuit. The TS881 voltage comparator compares *V_ref_* with the envelope detection output V_ED_. A DC–DC converter drive signal is generated through this comparison.

The maximum power point voltage is denoted as *V_MPP_*. When *V_rec_* exceeds *V_MPP_*, *V_ref_* surpasses *V_ED_*. A high-level output is triggered by this condition. The DC–DC converter is activated to pull down *V_rec_*. Power is supplied to the load by the DC–DC converter. Conversely, when *V_MPP_* is not reached, the DC–DC converter remains inactive. *V_rec_* rises gradually. Power is drawn directly from the rectified voltage by the load. The stable operation of the energy management circuit at the maximum power point is ensured by this mechanism.

**Figure 11:**
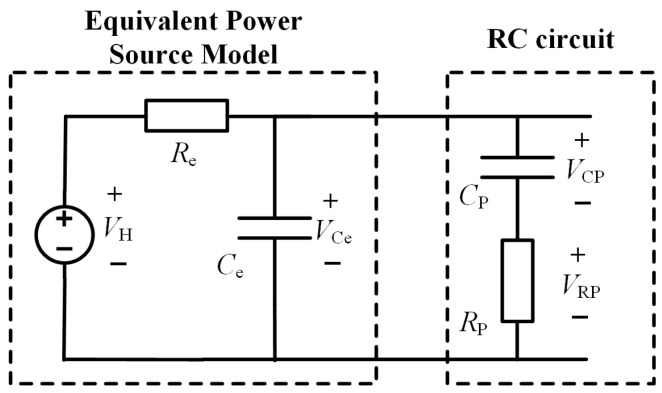
Equivalent circuit model.

Rectification is completed by the energy harvester. A series RC circuit is connected in parallel afterward. This RC circuit is composed of capacitor *C_P_* and resistor *R_P_*. The charging characteristics of capacitor *C_P_* are utilized. The maximum power point voltage *V_MPP_* is tracked through these characteristics. Maximum power point tracking control for the energy harvester is thereby achieved. The topology of the RC circuit (connected in parallel with the energy harvester) can be modeled as an equivalent circuit. This equivalent circuit is shown in Figure 11.

**Figure 12:**
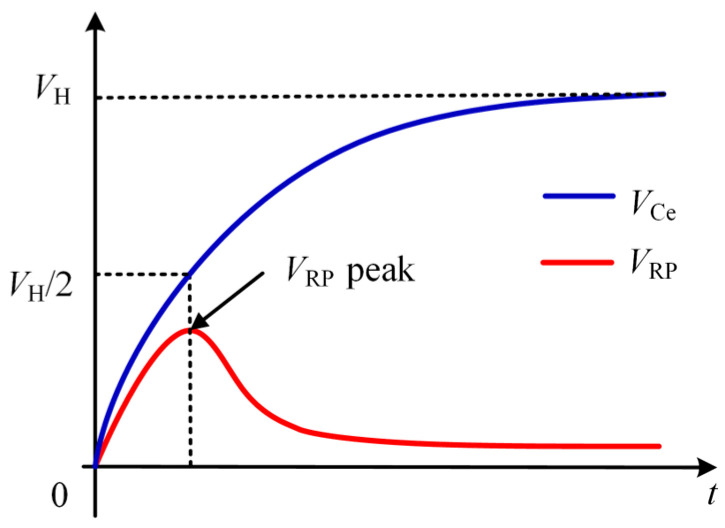
Implementing the MPPT principle using an RC circuit.

Its open-circuit voltage is denoted as *V_H_*. The equivalent resistance is represented by Re. The equivalent capacitance is denoted as *C_e_*. The time constant is represented by *τ_e_*. Within the RC circuit, *V_CP_* and V*_RP_* represent the voltages across capacitor *C_P_* and resistor *R_P_*, respectively. These two components are introduced in the circuit. The time constant parameter is appropriately set in the RC circuit. This setting maximizes the output voltage *V_Ce_* to the maximum power point voltage *V_MPP_*. When this is achieved, *V_RP_* reaches its peak value. The waveform of this circuit is shown in Figure 12.

The output voltage *V_RP_* in the RC circuit can be expressed by the following equation, as observed from this model:

(20) $$V_{RP}\left(t\right)=\;\left|V_{H}\right|[e^{-\frac{t}{\tau_{P}}}-e^{-(\frac{t}{\tau_{e}}+\frac{t}{\tau_{P}})}].$$

The energy harvesting system operates at its maximum power point when *V_Ce_* = *V_MPP_* = *V_H_*/2. From this condition, it can be derived that:

(21) $$1-e^{-\frac{t}{\tau_{e}}}\;=\;\frac{1}{2},\;t=\tau_{e}\ln2.$$

At this point, the output voltage *V_RP_* in the RC circuit reaches its peak value. This is expressed as:

(22) $$\frac{dV_{RP}(t)}{dt}\;=\;\left|V_{H}\right|\left[-\frac{t}{\tau_{P}}e^{-\frac{t}{\tau_{P}}}+\left(\frac{t}{\tau_{e}}+\frac{t}{\tau_{P}}\right)e^{-\left(\frac{t}{\tau_{e}}+\frac{t}{\tau_{P}}\right)}\right]=\;0.$$

It follows that:

(23) $$\tau_{e}\;=\;\tau_{P},t\;=\;\tau_{e}\ln2\;=\;\tau_{P}\ln2.$$

The time constant of the capacitor C_e_ charging process equals the time constant of the RC circuit when *τ_e_* = *τ_p_*. Under this condition, the equivalent capacitor voltage *V_Ce_* = *V_MPP_* = *V_H_*/2. Simultaneously, the output voltage V_RP_ of the RC circuit reaches its peak value. Therefore, MPPT control of the energy harvester can be achieved by tracking the peak voltage of *V_RP_*.

## 5. Experiment on Non-Invasive Magnetic Field Energy Harvesting Device

### 5.1. Prototype Design

**Figure 13:**
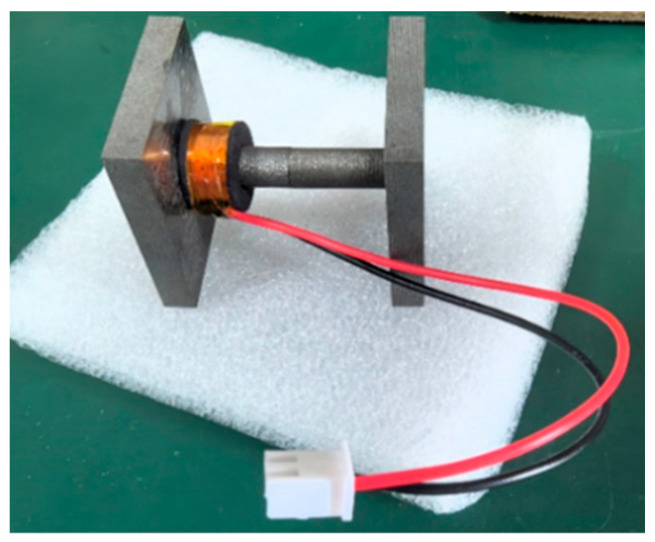
Non-intrusive magnetic field energy harvester prototype.

A non-invasive magnetic field energy harvester prototype is designed in this paper, shown in Figure 13. An H-shaped silicon steel 27Q100 magnetic core is employed. A conventional spiral coil is used as the energy harvesting coil. The total number of turns is 1000.

### 5.2. Experimental Results

**Figure 14:**
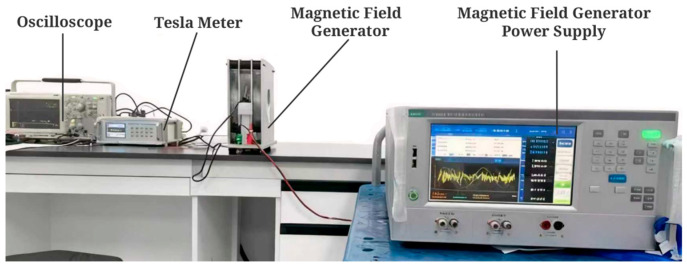
Energy harvester coil power experimental setup layout.

The experimental setup is shown in Figure 14. A 50 Hz alternating magnetic field with adjustable amplitude is produced by a magnetic field generator. Fifty Hz sinusoidal excitation is adopted in the test, and full consistency with the power-frequency leakage magnetic field is achieved by this excitation. The leakage magnetic field is generated by stator windings of actual grid-connected hydroelectric generating units, stipulations on the 50 Hz-rated frequency of the power system are complied with by this excitation, and the corresponding stipulations are specified in the national standards of the People’s Republic of China. An adjustable magnetic field range of 70–130 Gs is set in the test, and real leakage flux intensity around the hydro unit stator core under operating conditions is fully covered by this range. Real-time magnetic field strength is measured by a Teslameter. A standardized magnetic field environment is thereby established.

**Figure 15:**
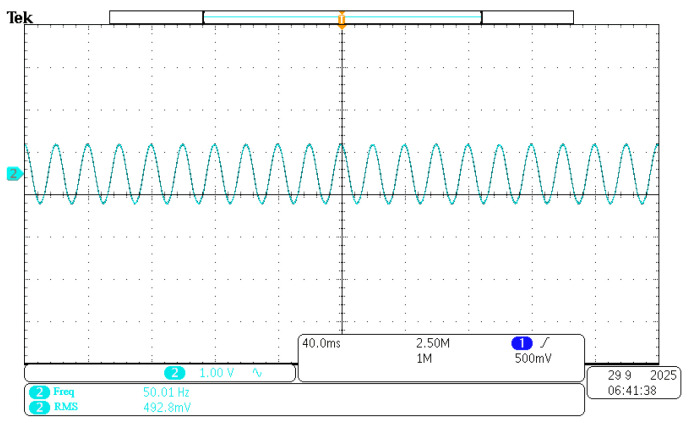
Fifty Gs Teslameter BNC connected to an oscilloscope to measure the magnetic field waveform.

The accuracy of the experimental magnetic field must be ensured. The output of the magnetic field generator is calibrated using a Teslameter for this purpose. A 50 Hz, 50 Gs RMS alternating magnetic field is taken as an example. The power supply is adjusted to generate this field using the Helmholtz coil. The measured value of 50 Gs meets the expected requirements. The 3 V voltage at its BNC port corresponds to the 300 Gs range. A sine wave is displayed by the oscilloscope, shown in Figure 15. The experimental requirements are met.

#### 5.2.1. No-Load Test for Energy Extraction Coils

**Figure 16:**
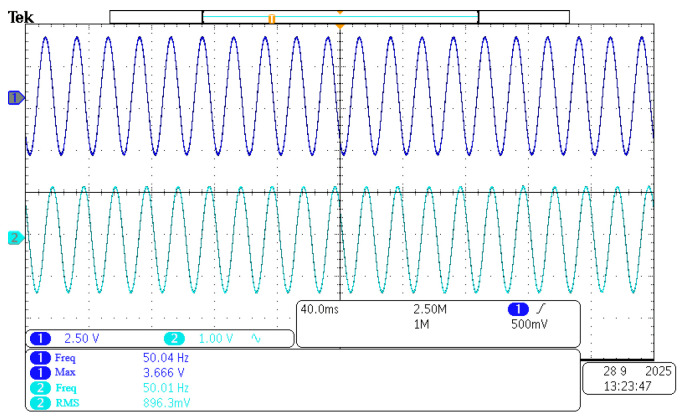
No-load output waveform of 1000-turn energy harvesting coil at 90 Gs magnetic field strength.

**Figure 17:**
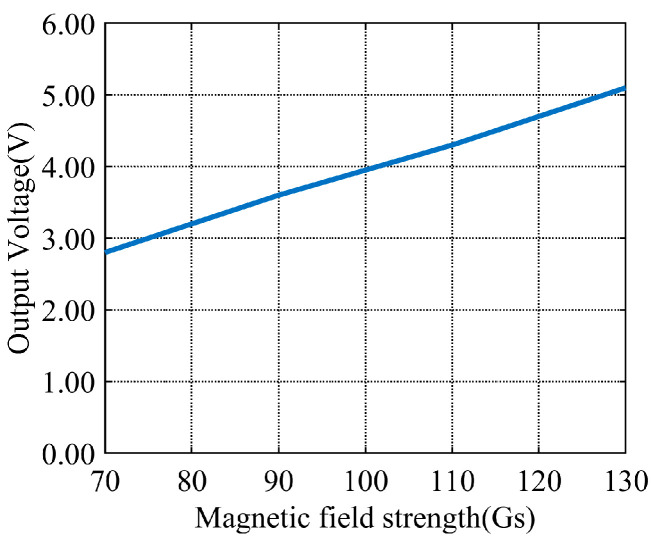
Fitted relationship between magnetic field strength and no-load output voltage curves.

The energy harvesting device is placed on the experimental bench. The bench is inside the magnetic field generator. The power supply of the magnetic field generator is adjusted. Experimental environments with varying magnetic field strengths are created. The output of the harvesting coil is tested under these environments. Ninety Gs is taken as an example. The experimental results are shown in Figure 16. The magnetic field waveform output from the Teslameter’s BNC port is represented by the green curve. The open-circuit voltage waveform output from the energy harvesting device is shown by the blue curve. Under these magnetic field conditions, an open-circuit output amplitude of approximately 3.6 V is produced by the single coil. The frequency is a 50 Hz alternating current. A fitted curve is also plotted. It illustrates the relationship between magnetic field strength and output voltage. This curve is shown in Figure 17.

The magnitude of the open-circuit voltage output from the energy harvesting device is proportional to the magnetic field strength. This indicates that magnetic field energy from the environment can be effectively captured by the harvesting coil. This energy is converted into alternating current.

#### 5.2.2. Load Test for Energy Extraction Coils

**Figure 18:**
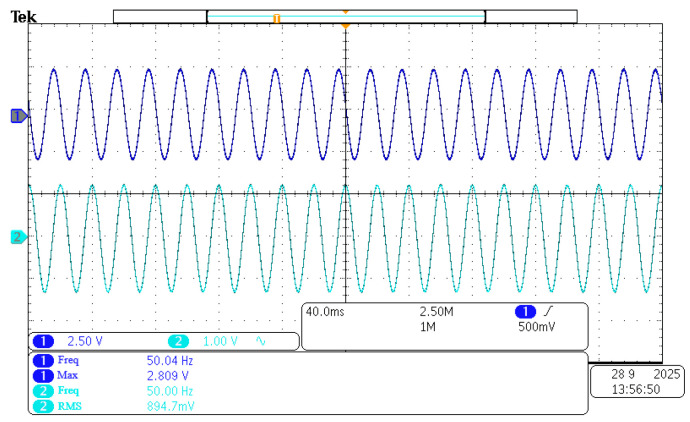
Loaded output waveform of 1000-turn energy harvesting coil at 90 Gs magnetic field strength.

**Figure 19:**
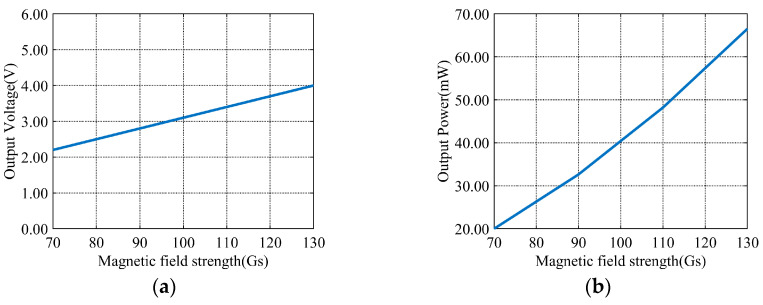
Fitted relationship between magnetic field strength, output voltage, and output power curves. (**a**) Output voltage under load. (**b**) Output power under load.

The aforementioned no-load experiments provide a basis. A 240 Ω load resistor is connected. It is used to simulate the output performance under actual operating conditions. Ninety Gs is taken as an example. The experimental results are shown in Figure 18. Under this magnetic field environment, an alternating voltage is output by the energy harvesting device. The amplitude is approximately 2.8 V, and the frequency is 50 Hz. Simultaneously, a fitted curve is plotted. It illustrates the relationship between magnetic field strength and output voltage. This curve is shown in Figure 19a.

Favorable output characteristics are maintained by the energy harvesting device under load conditions. However, the voltage level decreases due to load voltage division. A fitted curve is plotted. It illustrates the relationship between magnetic field strength and output power. The curve shown in Figure 19b is obtained. Across different magnetic field environments tested, an average power exceeding 10 mW is delivered by the harvesting coil. A peak power approaching 66 mW is also achieved. This demonstrates excellent energy harvesting capability.

#### 5.2.3. Joint Testing of Energy Harvesting Coil and Energy Management Circuit

**Figure 20:**
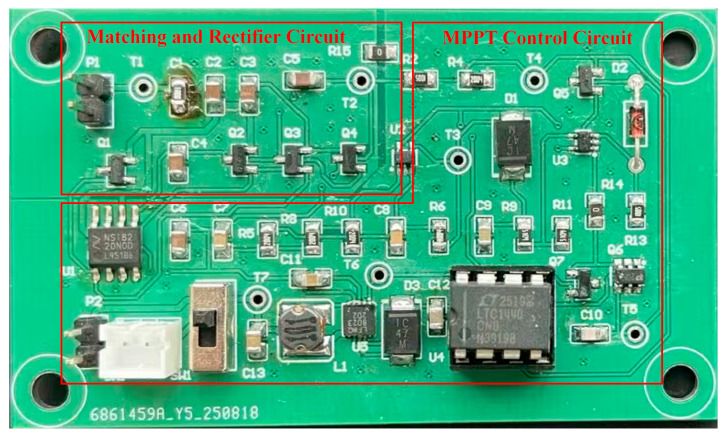
Energy management circuit PCB prototype.

**Figure 21:**
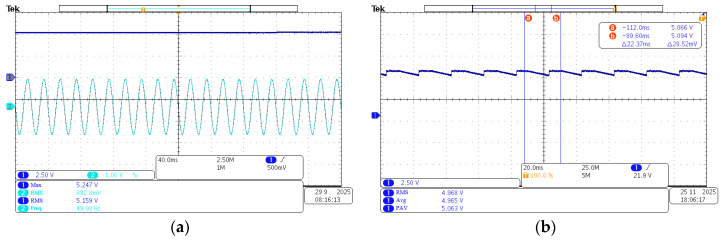
No-load and loaded output waveforms of energy harvesting coil and energy management circuit at 90 Gs magnetic field strength. (**a**) No-load; (**b**) under load.

The aforementioned energy harvesting coil tests provide a basis. An energy management circuit is integrated, shown in Figure 20. Real-world operating conditions are simulated by this integration. A magnetic field strength of 90 Gs is taken as an example. The no-load output of the energy harvesting coil and energy management circuit is tested. The experimental results are shown in Figure 21a. The combined maximum output voltage of the system is approximately 5.24 V. The root mean square (RMS) voltage is approximately 5.15 V. Simultaneously, the system’s combined output under load is shown in Figure 21b. The average voltage of the combined output is approximately 4.96 V. The root mean square (RMS) voltage is approximately 4.96 V.

The results demonstrate that stable output voltage can be achieved by the magnetic field energy harvesting system. This system is constructed using an energy harvesting coil and an energy management circuit. The stable output is achieved under specific magnetic field strength conditions. Furthermore, Maximum Power Point Tracking (MPPT) control is successfully implemented by the system under load conditions. Excellent power regulation performance and voltage stability are exhibited. The full 70–130 Gs-tested magnetic field range is covered in the test. Stable DC output is achieved by the system with the proposed MPPT circuit, and impedance mismatch loss is eliminated by this system. An average output power 40–50% higher than that of the direct rectifier system without MPPT is obtained by the proposed system, and the high-efficiency energy conversion capability of the designed circuit is quantitatively verified by the test results.

## 6. Conclusions

A non-invasive magnetic field energy harvesting solution is proposed in this paper. It is designed for the stator core region of hydroelectric units. Core and coil parameters are optimized to support this solution. Theoretical analysis, simulation optimization, and experimental validation are conducted. The effectiveness and practicality of the system are confirmed by experimental validation. Specified test conditions are set as 50 Hz power frequency and a 70–130 Gs magnetic field. Full matching with the actual hydropower operating environment is achieved by this magnetic field range. Competitive energy harvesting performance is presented by the tested system. No-load output voltages ranging from approximately 2.8 V to 5.1 V and loaded output voltages ranging from approximately 2.2 V to 4.0 V are measured for the single coil. A maximum output power of about 66 mW is achieved by the system, along with a power density of approximately 6.5 mW/cm^3^.

Real hydropower operating conditions are set as the target application scenario; stable DC output is expected to be maintained by the system, and continuous and peak power requirements of stator condition monitoring sensors are fully satisfied by this stable output. The long-term vibration and leakage flux fluctuation of operating units are adapted to by the system, and the corresponding adaptation capability is realized through the optimized mechanical structure and high-efficiency MPPT control circuit. Long-term maintenance-free operation is supported by the system, and this operation support is achieved via the non-intrusive battery-free design. The drawbacks of traditional power supply solutions are avoided by this design. Stable output voltage is delivered by the integrated system. Maximum power tracking control is implemented by the system.

## Data Availability

The original contributions presented in this study are included in the article. Further inquiries can be directed to the corresponding author.
